# Clinical translation of a patient-specific scaffold-guided bone regeneration concept in four cases with large long bone defects

**DOI:** 10.1016/j.jot.2022.04.004

**Published:** 2022-06-16

**Authors:** Markus Laubach, Sinduja Suresh, Buddhi Herath, Marie-Luise Wille, Heide Delbrück, Hatem Alabdulrahman, Dietmar W. Hutmacher, Frank Hildebrand

**Affiliations:** a Department of Orthopaedics, Trauma and Reconstructive Surgery, RWTH Aachen University Hospital, Pauwelsstraße 30, 52074, Aachen, Germany; b Australian Research Council (ARC) Training Centre for Multiscale 3D Imaging, Modelling, and Manufacturing (M3D Innovation), Queensland University of Technology, Brisbane, QLD, 4000, Australia; c Centre for Biomedical Technologies, School of Mechanical, Medical and Process Engineering, Queensland University of Technology, Brisbane, QLD, 4059, Australia; d Max Planck Queensland Center for the Materials Science of Extracellular Matrices, Queensland University of Technology, Brisbane, QLD, 4000, Australia; e ARC Training Centre for Cell and Tissue Engineering Technologies, Queensland University of Technology (QUT), Brisbane, QLD, 4000, Australia

**Keywords:** additive manufacturing, bone, defect, non-union, polycaprolactone, scaffold

## Abstract

**Background:**

Bone defects after trauma, infection, or tumour resection present a challenge for patients and clinicians. To date, autologous bone graft (ABG) is the gold standard for bone regeneration. To address the limitations of ABG such as limited harvest volume as well as overly fast remodelling and resorption, a new treatment strategy of scaffold-guided bone regeneration (SGBR) was developed. In a well-characterized sheep model of large to extra-large tibial segmental defects, three-dimensional (3D) printed composite scaffolds have shown clinically relevant biocompatibility and osteoconductive capacity in SGBR strategies. Here, we report four challenging clinical cases with large complex posttraumatic long bone defects using patient-specific SGBR as a successful treatment.

**Methods:**

After giving informed consent computed tomography (CT) images were used to design patient-specific biodegradable medical-grade polycaprolactone-tricalcium phosphate (mPCL-TCP, 80:20 ​wt%) scaffolds. The CT scans were segmented using Materialise Mimics to produce a defect model and the scaffold parts were designed with Autodesk Meshmixer. Scaffold prototypes were 3D-printed to validate robust clinical handling and bone defect fit. The final scaffold design was additively manufactured under Food and Drug Administration (FDA) guidelines for patient-specific and custom-made implants by Osteopore International Pte Ltd.

**Results:**

Four patients (age: 23–42 years) with posttraumatic lower extremity large long bone defects (case 1: 4 ​cm distal femur, case 2: 10 ​cm tibia shaft, case 3: complex malunion femur, case 4: irregularly shaped defect distal tibia) are presented. After giving informed consent, the patients were treated surgically by implanting a custom-made mPCL-TCP scaffold loaded with ABG (case 2: additional application of recombinant human bone morphogenetic protein-2) harvested with the Reamer-Irrigator-Aspirator system (RIA, Synthes®). In all cases, the scaffolds matched the actual anatomical defect well and no perioperative adverse events were observed. Cases 1, 3 and 4 showed evidence of bony ingrowth into the large honeycomb pores (pores >2 ​mm) and fully interconnected scaffold architecture with indicative osseous bridges at the bony ends on the last radiographic follow-up (8–9 months after implantation). Comprehensive bone regeneration and full weight bearing were achieved in case 2 ​at follow-up 23 months after implantation.

**Conclusion:**

This study shows the bench to bedside translation of guided bone regeneration principles into scaffold-based bone tissue engineering. The scaffold design in SGBR should have a tissue-specific morphological signature which stimulates and directs the stages from the initial host response towards the full regeneration. Thereby, the scaffolds provide a physical niche with morphology and biomaterial properties that allow cell migration, proliferation, and formation of vascularized tissue in the first one to two months, followed by functional bone formation and the capacity for physiological bone remodelling. Great design flexibility of composite scaffolds to support the one to three-year bone regeneration was observed in four patients with complex long bone defects.

**The translational potential of this article:**

This study reports on the clinical efficacy of SGBR in the treatment of long bone defects. Moreover, it presents a comprehensive narrative of the rationale of this technology, highlighting its potential for bone regeneration treatment regimens in patients with any type of large and complex osseous defects.

## 1 Introduction

Bone is a dynamic, vascularized tissue which constantly remodels to adapt to mechanical demands [1]. Bone tissue healing, as a regenerative process, is initiated in response to injury and involves multiple well-orchestrated steps. Developments in surgical techniques, implant design and perioperative management have significantly improved the treatment of complex fractures and other skeletal defects caused by trauma, disease, developmental deformity, and tumour resection. Nonetheless, treatment of (large) long bone defects is complex, and the risk of malunion, delayed union and non-union remains high, with these problems frequently resulting in persistent and challenging osseous defects, which are associated with reduced quality of life and significant health care costs [[2], [3], [4]]. Importantly, limb-threatening trauma involving the challenging treatment of bone defects constitutes a major event in a patient's life [5].

While smaller-sized defects (<5 ​cm) are often treated with autologous bone graft (ABG) alone [6,7], ABG used for reconstruction of larger long bone defects (≥5 ​cm) is associated with an increased risk of resorption [8] and structurally and functionally compromised regenerated bone in the reconstructed segment [9]. Historically, surgical methods for the treatment of large long bone defects have included vascularized free bone transfer [[10], [11], [12]] or the Ilizarov intercalary bone transport method [13]. However, while all of these methods are technically demanding and are associated with high complication rates [14,15], the Ilizarov method (distraction osteogenesis) in particular is characterised by a long duration of external fixation with marked patient discomfort and requiring high patient compliance [16,17]. For the last two decades, the induced membrane technique (IMT), first described by Alain Masquelet, has frequently been used as an alternative approach in cases of large long bone defects [[18], [19], [20], [21], [22]]. IMT involves a two-stage approach with implantation of a polymethylmethacrylate (PMMA) spacer in the bone defect to induce the formation of a (pseudo) Masquelet-membrane followed by an attempt to reconstruct the osseous defect in a second surgery, four to eight weeks after the first operation, using an ABG. Despite considerable clinical success rates for IMT, bone healing often requires multiple additional interventions, and a failure rate of 10%–15% has been reported [[23], [24], [25]].

In order to account for the challenges associated with the use of ABG (e.g. limited graft volume, graft resorption), there has been interest of late in the use of naturally derived and/or synthetic bone graft substitutes. However, the application of allografts is associated with very variable osteoinductive properties, high non-union rates and potential disease transmission [26,27]. Internal repair by means of neovascularization and bone graft replacement with new bone seems limited to the superficial surface and the ends of the allograft [28,29]. Furthermore, fractures of the allograft itself, particularly when used for larger defects, have been reported [30]. Moreover, the high cost of allografting has further spurred the development of other strategies, including the use of synthetic bone substitutes [31]. However, synthetic bone graft substitutes, such as hydroxyapatite and/or tricalcium phosphate, can only be applied in combination with ABG at a maximum ratio of 1:1 to 1:3 due to high non-union rates [26,[32], [33], [34], [35]]. Thus, the clinical applicability of allografts and synthetic bone substitutes for bone defects is often limited.

The concept of scaffold-guided bone regeneration (SGBR) is rooted in the application of an additively manufactured biodegradable highly porous implant (“scaffold”) that has two functions: acting as a carrier for growth factors such as recombinant human bone morphogenetic proteins (rhBMP) 2 or 7 and/or bone grafts, and physically guiding tissue regeneration, acting as a template for both the transplanted bone graft and the host cells to ultimately result in tissue ingrowth and physiological remodelling [36]. Particularly in response to the highly dynamic tissue processes during the first three to six months of bone regeneration, the use of scaffolds provides an appropriate physical environment, as this is typically associated with disturbed local soft tissue integrity, disruption of normal vascular function, and distortion of bone marrow architecture due to surgery. Extravasation (bleeding) at the implantation site of the scaffold is contained by the surrounding tissue and develops into hematoma, neovascularization and development of osteogenic tissue and later bone remodeling. As such, specifically slow degrading scaffolds provide a temporary architecture and a suitable environment to facilitate bone regeneration [37].

SGBR rooted in combining 3D-printed biodegradable composite scaffolds with different types of ABGs, rhBMP-2 or rhBMP-7 has been successfully applied to treat large to extra-large long bone defects in several comprehensive preclinical large animal studies [[38], [39], [40], [41], [42]]. Furthermore, recent case reports indicate the successful translation of patient-specific SGBR in patients with bone loss of traumatic or tumorous origin for individual healing attempts, including the capacity for preservation of the lower extremities [[43], [44], [45]]. Building on both the comprehensive preclinical data sets and the first clinical case reports, in this case series, we demonstrate in great detail the versatility of the patient-specific SGBR concept for the treatment of posttraumatic long bone defects.

## 2 Methods and materials

**Fig. 1:**
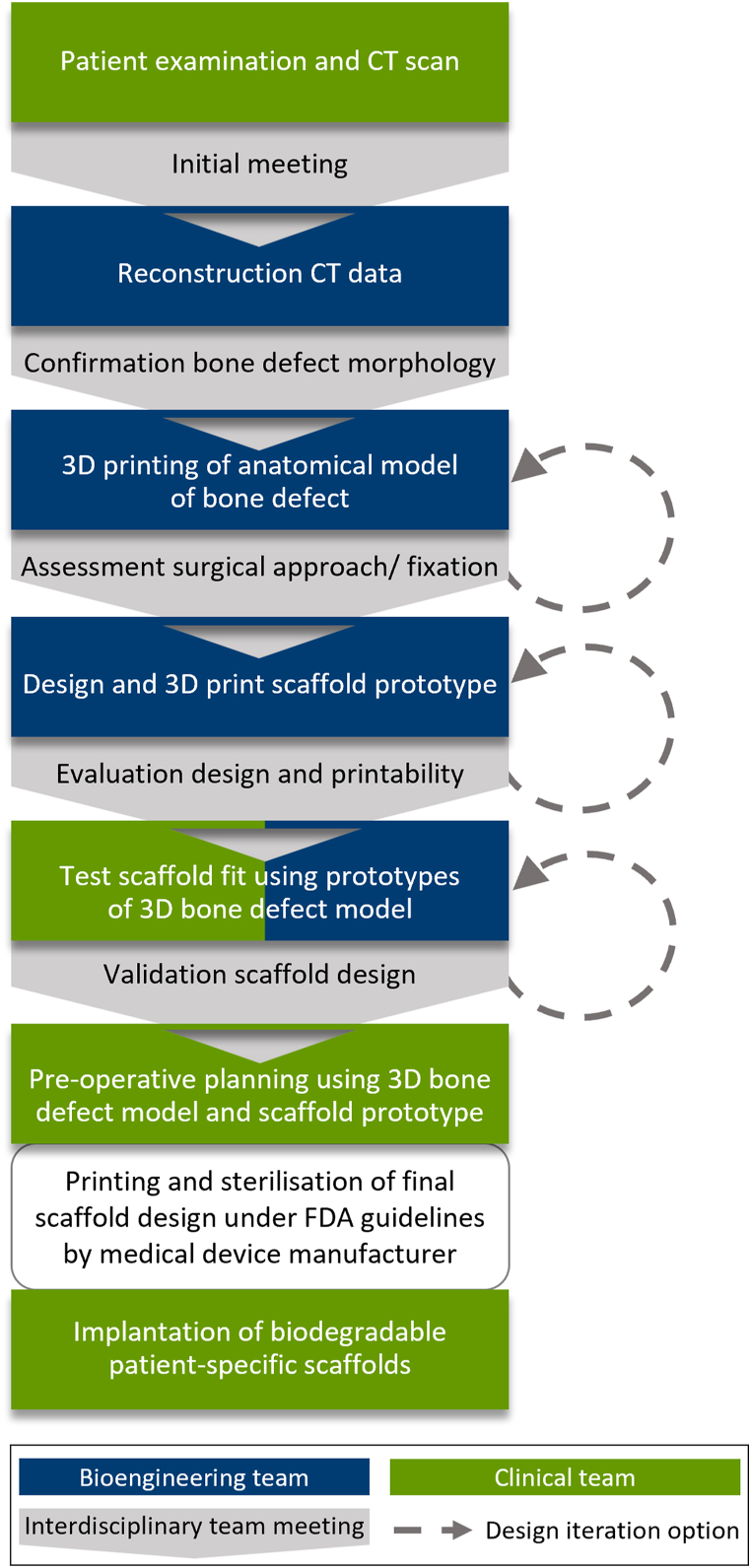
Workflow of the interdisciplinary process for the development and manufacturing of the patient-specific biodegradable scaffolds.

SGBR, in the context of developing a patient-specific treatment concept, is an interdisciplinary and iterative process with prototype verification of both the anatomical model of the defect and the implant design (scaffold) at every stage. In the interdisciplinary meetings between clinicians and engineers, the entire treatment concept including surgical access and the intended scaffold fixation methods is discussed and simulated (Fig. 1).

### 2.1 Design process and manufacturing

The first step is to CT scan the patient according to standard scanning protocols. Notably, higher resolution than that normally clinically recommended for the CT scan will improve the accuracy of both the anatomical model and the scaffold design. Subsequently, the CT image data segmentation is performed on specialised software such as the Mimics Suite (Materialise, Leuven, Belgium) to extract a 3D model of the defect. It is recommended to add any fitting of surgical fixation methods (osteosynthesis plates or intramedullary nails) to the anatomical defect model prior to designing the scaffold. The defect model is then exported as an Standard Tessellation Language (STL) (surface mesh format) file and imported into a mesh manipulation software, such as Materialise 3-matic, Geomagic (3D Systems, USA) or Autodesk Meshmixer (Autodesk Inc., USA). It is strongly recommended to use 1 to 1 anatomical models of the bone defect site with and without the planned (internal) fixation to physically simulate the fit and accuracy of the scaffold.

**Fig. 2:**
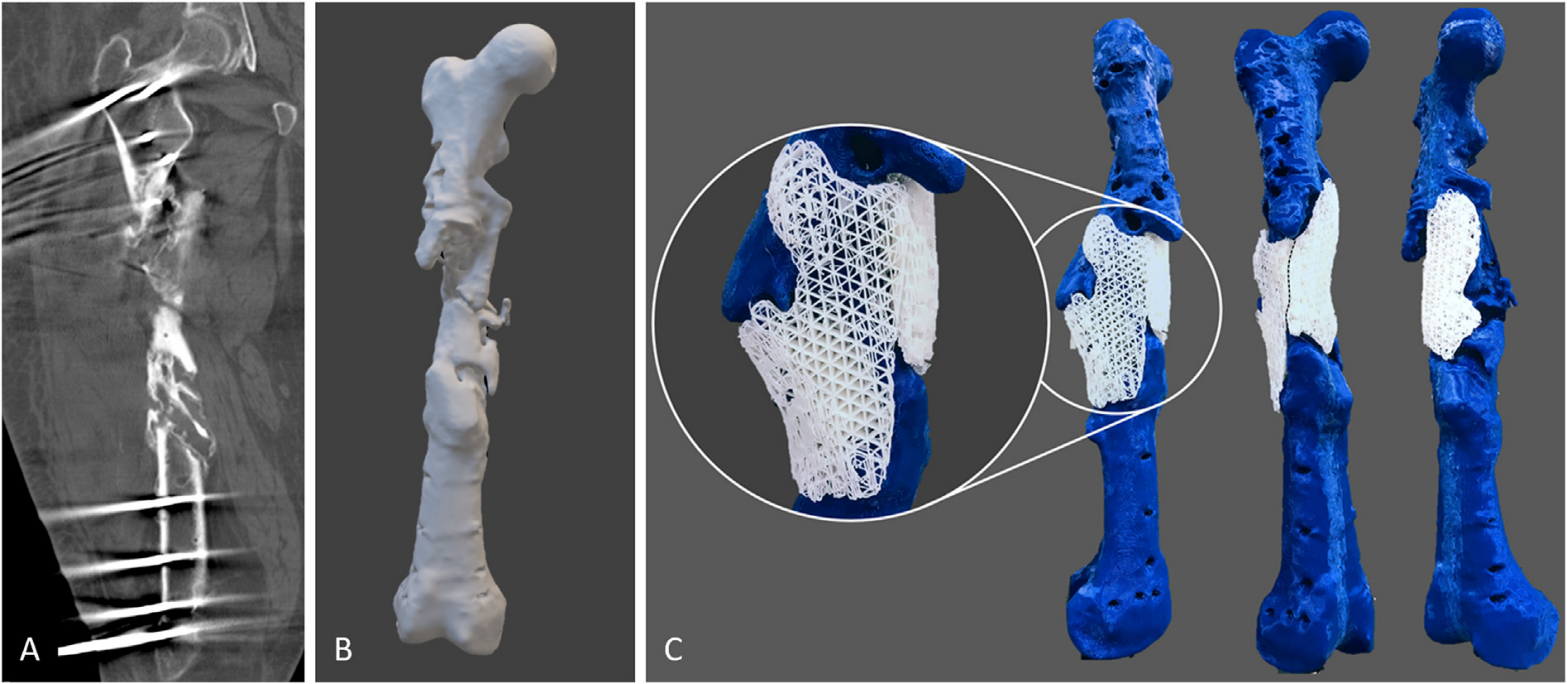
Schematic depiction of the development steps towards the optimized design of patient-specific biodegradable scaffolds for use in complex large bone defects. Typically, the hospital undertakes a CT scan and provides the team designing the scaffold with the acquired image data. Cross-sectional images of the CT scan (A) are segmented and converted into STL files. Based on the information stored in the STL file, the surface geometry of the 3D model (B) and the defect-fitting scaffolds are 3D-printed (C, exemplary prototypes of the modular, two-part mPCL-TCP scaffold of case 3 fitting the complex femoral bone defect). Modular design, with large pore sizes of 0.8–3 ​mm for incorporation of bone graft (see magnification in C), allowed for unilateral surgical access with placement of lateral scaffold first followed by the medial scaffold (black dashed line indicates the contact point of the two scaffolds).

The scaffold is designed digitally on a mesh manipulation software: a base 3D shape is created, modified slightly to fit the bone void, and strategically placed to cover and fill the defect site. In the case of segmental defects and regularly shaped voids, it is common practice to create a 3D model of the contralateral region and to use a mirrored version of this model to design the scaffold. However, in bone defects where more complex geometry matching is required, reconstruction will involve more manual intervention. Once the base shape is placed correctly, a Boolean subtraction is performed (base shape minus the defect model). This sculpts the geometry of the defect site on the base shape. The external surface of the sculpted base shape is then manually sculpted (using a wide array of digital mesh sculpting tools) around the defect to ensure adequate coverage, close fit and appropriate surface thickness throughout the scaffold so that stability is maintained. The result is two 3D models: the defective bone and a scaffold part that fits it, analogous to two 3D puzzle pieces that fit together. Fig. 2 shows a schematic of the applied design process. Multiple scaffold parts may be required to facilitate user friendly insertion by the surgeon and take into account the surgical access, incision size and clearance from the stabilisation device, all factors which are addressed during the interdisciplinary team meetings. The individually planned defect stabilization method, such as an intramedullary nail or a plate osteosynthesis influences the overall scaffold design. In addition, the scaffold parts should also be capable of being inserted into the defect void without being obstructed by the existing bone structure.

Furthermore, if fixation of the scaffolds to the host bone is required, non-porous flanges are added to the scaffold parts to fit over locations deemed biomechanically optimal on the host bone. Flanges can be created by expanding the 3D mesh at that point and performing another simple Boolean subtraction with the host bone to ensure patient-specific fit. Screws may be used to secure scaffolds onto the host bone through these flanges. To ensure that the geometrically matched scaffolds are capable of complete insertion with proper fit and to assist in pre-operative planning, prototypes of the defect model and the scaffolds are 3D printed and tested by the interdisciplinary team. Lastly, the finalised scaffold designs are shared with the medical device manufacturer Osteopore International Pte Ltd. (MedTech Hub, Singapore) for fabrication of the patient-specific scaffolds by using medical-grade polycaprolactone-tricalcium phosphate (mPCL-TCP, 80:20 ​wt%). A melt extrusion 3D printing technology is used to manufacture an alternating rectilinear infill pattern of 0°, 60° and 120° creating triangular pores with a size of 2–3 ​mm. Briefly, in accordance with Food and Drug Administration (FDA) guidelines, the patient-specific scaffolds were manufactured in a clean-room environment adhering to International Organization for Standardization (ISO) 14644. Further, under the ISO 13485-compliant quality management system of Osteopore all implants were packed ISO 11607 compliant and sterilized with gamma irradiation according to ISO 11137 guidelines.

## 3 Results

Four patients with posttraumatic long bone defects are reported hereinafter, all of whom are characterised by a complex course of therapy. After receiving detailed spoken and written information, all patients explicitly provided spoken and written informed consent to agree to the suggested treatment, including the implantation of mPCL-TCP scaffolds. Case characteristics and surgical treatment strategies applied to achieve bone healing are presented in Table 1. As part of the complex treatment course, the patients were treated with two-stage IMT and received mPCL-TCP scaffolds for an individual healing attempt during the second surgery of the IMT. The patient-specific scaffold prototypes for the four cases are displayed in Supplement 1. The pore size may vary depending on the exact geometry of the bone defect; however, 2 ​mm was respected as minimum. In all cases the scaffolds were used in combination with ABG. Therefore, the scaffolds designed with sufficiently large pores were carefully loaded manually with fresh bone graft. Particular care was taken to ensure that the pores were thoroughly and homogeneously loaded with bone graft, and in this regard the ease of access to the scaffolds from all sides proved beneficial. In the cases presented, satisfactory loading of the scaffolds with ABG was achieved in less than 5 ​min, and the properly sized pores combined with the suitable viscosity of the bone graft ensured its retention in the pore architecture, notably also during handling and implantation.

**Table 1:** Case characteristics and treatment strategies applied to achieve bone healing.

Case number (patient age)	Anatomical site (index trauma)	Bone defect morphology at the time of scaffold implantation (defect volume∗)
**Case 1 (23 years)**	Distal femur metaphysis (grade III open fracture)	Extensive non-union with bone shortening causing a leg length discrepancy of −4 cm (73.67 ​cm^3^∗∗)
0 months after index trauma	External fixator and treatment of local infection	
2 months after index trauma	Procedural change to less invasive stabilization system (LISS, Synthes®) plate	
6 months after index trauma	Open biopsy and initiation of IMT	
7 months after index trauma	Replacement of the LISS plate by a longer Non-Contact Bridging (NCB, Zimmer®) plate plus implantation of a Locking Compression Plate (LCP, Synthes®) medially along with insertion of a tubular mPCL-TCP scaffold loaded with Cerament G® (BONESUPPORT AB) and RIA ABG	
8 months after scaffold implantation	Unrestricted pain-free ability to walk without the support of assistive devices; advanced bony fusion on radiographic imaging	
**Case 2 (27 years)**	Tibia shaft (grade III open fracture)	Extra-large 10 ​cm-sized segmental defect (47.13 ​cm^3^)
0 months after index trauma	External fixator and treatment of local infection	
0–5 months after index trauma	Partial resection of the tibia during a complicated course of treatment	
6 months after index trauma	Implantation of an Orthofix® external fixator (TrueLok™ Ring Fixation System) and initiation of IMT	
7 months after index trauma	Replacement of PMMA spacer by a tubular scaffold loaded with RIA ABG and Cerament G® (BONESUPPORT AB) and supplemented with rhBMP-2	
12 months after scaffold implantation	Replacement external fixator with medial angular stable plate	
19 months after scaffold implantation	Bony fusion on CT scan	
23 months after scaffold implantation	Implant removal; pain-free full weight bearing within 2 weeks	
**Case 3 (42 years)**	Femur shaft (complex multi-fragmentary fracture)	Complex malunion (165.72 ​cm^3^)
0–5 months after index trauma	Initial treatment with external fixator and large fragment plate	
6 months after index trauma	Open biopsy with septic debridement and fistula revision	
6–7 months after index trauma	Removal of the atypically inserted plate, sequestrectomy, and exchange of the external fixator with a lateral femoral hybrid fixator (Orthofix®) as well as a Vacuum Assisted Closure (VAC) therapy including its regular exchanges	
19 months after index trauma	Implantation of modular (two parts) 3D-printed mPCL-TCP scaffolds loaded with ABG and combined with plate osteosynthesis	
6 months after scaffold implantation	Radiographically confirmed relevant osseous consolidation; pain-free full weight bearing using forearm crutches	
9 months after scaffold implantation	Radiographically confirmed progressing bony fusion	
**Case 4 (30 years)**	Distal tibia metaphysis (complex multi-fragmentary lower leg fracture)	Irregularly shaped large defect (29.89 ​cm^3^)
0–4 months after index trauma	External fixator (tibia) and small diameter intramedullary wire (fibula)	
5 months after index trauma	Open biopsy	
5 months after index trauma	Change external fixator to an Orthofix® ring fixator (TrueLok™ Ring Fixation System) and insertion of Cerament V® (BONESUPPORT AB) into the medullary cavity	
19 months after index trauma	Procedural change to intramedullary nail fixation	
20 months after index trauma	Early nail removal due to recurrent osteomyelitis	
21 months after index trauma	External fixator and initiation of IMT	
22 months after index trauma	Placement of LCP 3.5 (Synthes®) and implantation of two-part mPCL-TCP scaffold loaded with iliac crest and RIA ABG as well as Cerament V®	
7 months after scaffold implantation	Pain-free full weight bearing using forearm crutches for additional support	
8 months after scaffold implantation	Radiographically confirmed bone formation inside and outside the fully interconnected scaffold architecture	

∗ Bone defect volume was calculated by segmenting the CT image data and performing Boolean subtraction from an idealised intact bone volume.

∗∗The calculated defect volume is very likely an underestimate of the actual defect volume, as CT data was used for the calculation from a scan with the bone in impacted, shortened plate fixation.

### 3.1 Case 1

**Fig. 3:**
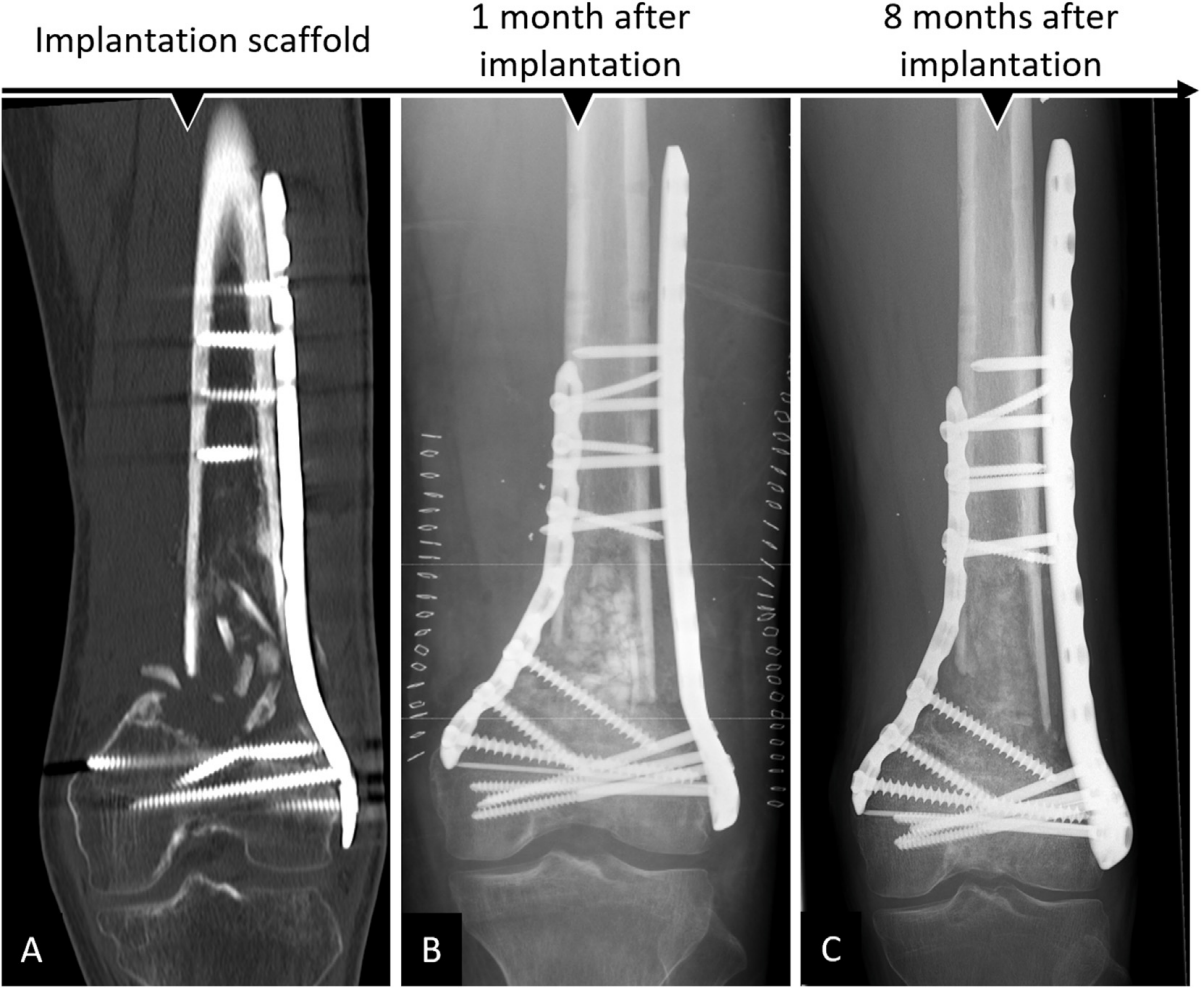
An mPCL-TCP scaffold loaded with ABG was used to treat femoral non-union and leg length discrepancy (- 4 ​cm) in a 23-year-old patient. The implanted LISS plate (Synthes®, A) was removed, and the patient received re-osteosynthesis using an NCB plate (nine holes, Zimmer®) laterally and LCP (eight-hole large fragment system, Synthes®) medially (B). At the short-term follow-up, delicate but adequate bone formation with full scaffold integration (C).

A 23-year-old patient presented with a non-union of the distal left femur and a clinically relevant leg length discrepancy (LLD, −4 ​cm) six months after sustaining an open (grade III) distal femur fracture. Initial injury treatment was ex domo using first an external fixator and then changing to a less invasive stabilization system (LISS, Synthes®) plate after successful treatment of a local infection. In our care, the initial treatment included an open biopsy of the femoral defect area and initiation of IMT, including implantation of a PMMA spacer. No microbial colonisation was revealed in the open biopsy and the patient received a tubular mPCL-TCP scaffold loaded with ABG harvested from the contralateral femur using the Reamer-Irrigator-Aspirator (RIA, Synthes®) device mixed with Cerament G® (BONESUPPORT AB, Lund, Sweden). As the LLD was also corrected, the LISS (Synthes®) plate on the lateral site was removed and a longer NCB (Non-Contact Bridging, Zimmer®) plate was implanted. In the same operation, to achieve additional stability, a Locking Compression Plate (Large Fragment System, Synthes®) was implanted medially. Eight months after scaffold implantation, the patient showed an unrestricted pain-free ability to walk without the support of assistive devices. Furthermore, plain radiographic imaging showed advanced bony fusion (Fig. 3).

### 3.2 Case 2

**Fig. 4:**
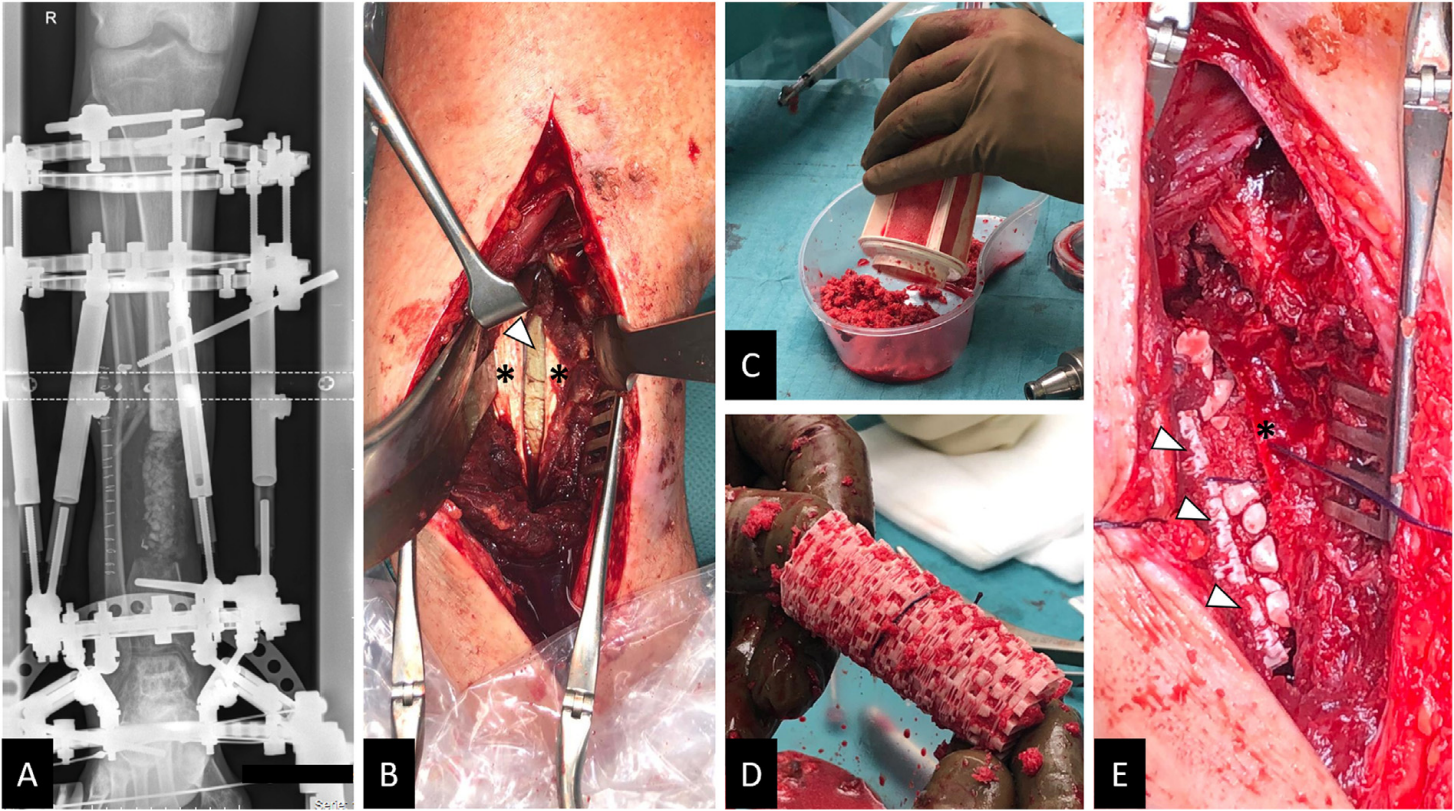
Treatment of extra-large tibial segmental (10 ​cm) defect with mPCL-TCP scaffold loaded with ABG and supplemented with rhBMP-2 (INFUSE® Bone Graft, Medtronic). An Orthofix® (TrueLok™ Ring Fixation System) was implanted to allow for full weight bearing, while an inserted antibiotic-impregnated PMMA spacer was used to initiate the IMT (A). In the second surgery of the two-stage IMT, the PMMA spacer (white triangle) is carefully removed after Masquelet-membrane (asterisks) incision (B). The RIA system (Synthes®) was used to harvest the ABG (C), which was then carefully inserted into the large-pored scaffold (D). After insertion of the scaffold loaded with ABG (white triangles) and supplemented with rhBMP-2 and Cerament G® in the segmental defect, the Masquelet-membrane (asterisk) was closed (E).

**Fig. 5:**
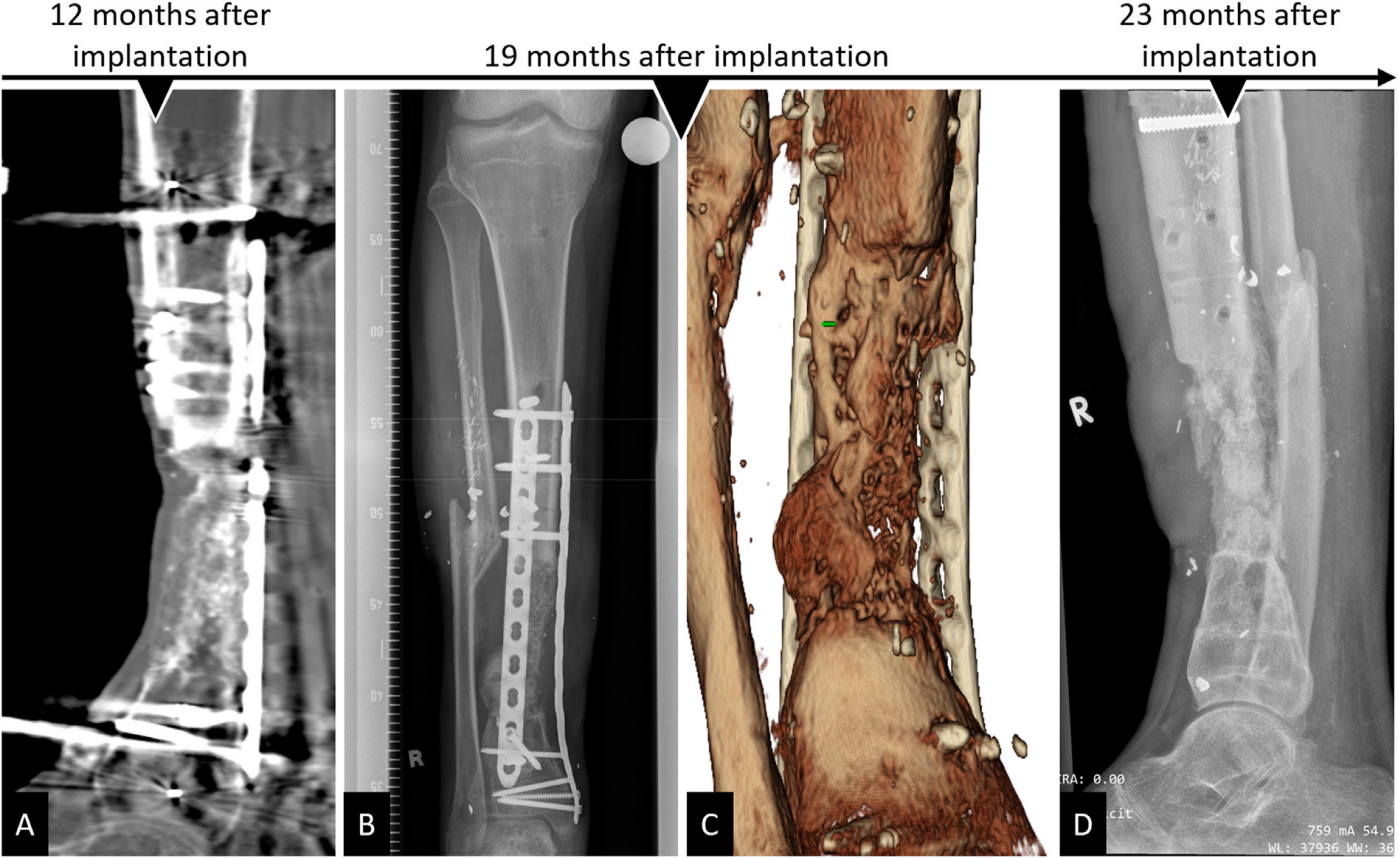
Complete bone regeneration achieved in an extra-large tibial segmental (10 ​cm) defect after implantation of an mPCL-TCP scaffold loaded with ABG and supplemented with rhBMP-2 (INFUSE® Bone Graft, Medtronic). Scaffold integration at the proximal and distal defect ends after one year (A) was observed, and an external fixator was exchanged with plate fixation. At 19 months after scaffold implantation, x-ray (B) and 3D reconstruction of the CT scan (C) showed bony fusion. At 23 months after scaffold implantation, functional reconstruction of the extra-large segmental defect was achieved and the osteosynthesis implants were removed (D).

A 27-year-old patient underwent partial resection of the right tibia during a complicated course of treatment, including recurrent osteomyelitis, after a III° open lower leg fracture. Six months after the initial trauma, the patient was transferred to our hospital for further therapy of a 10 ​cm-sized segmental defect. IMT was initiated after extensive debridement and lavage, and a modified external fixator from Orthofix® (TrueLok™ Ring Fixation System) was implanted, allowing immediate full weight bearing post-surgery. Supplement 2 details the surgical course of treatment before scaffold implantation. After antibiotic treatment, the second stage of the IMT technique was followed. The antibiotic-impregnated PMMA spacer was replaced by a tubular mPCL-TCP scaffold loaded with ABG harvested from the contralateral femur using the RIA system mixed with Cerament G® and supplemented with a bone morphogenetic protein-2 (12 ​mg)-impregnated collagen membrane (INFUSE® Bone Graft, Medtronic; Fig. 4). Two months after surgery, radiographic imaging showed minor dorsal scaffold dislocation, which was surgically addressed by fixation with an additional dorsal plate and by transplantation of additional ABG harvested from the iliac crest. Notably, during this revision surgery, there was no apparent evidence of inflammation in the area around the scaffold. Intraoperatively taken samples remained sterile. Eleven months after scaffold implantation, significant bone formation was observed, and the modified external fixator was replaced with an additional medial angular stable plate that was implanted in a minimally invasive way. Twenty-three months after scaffold implantation, a CT scan showed bony fusion and hence implant removal was indicated (Fig. 5). Following the implant removal, the patient had no pain and underwent full weight bearing within 2 weeks without any assistive devices.

### 3.3 Case 3

**Fig. 6:**
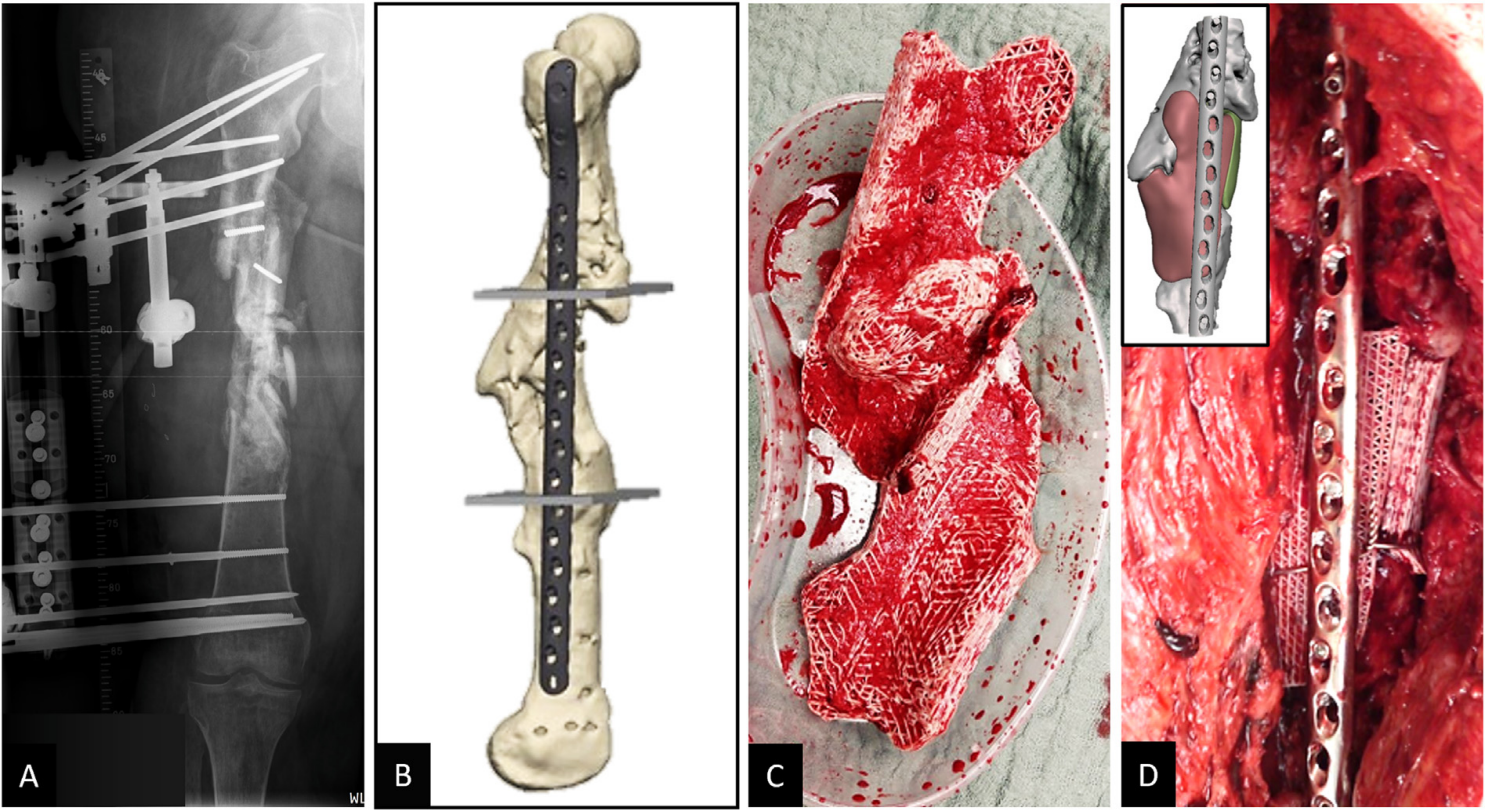
Treatment of large bone defect with complex malunion of the right femoral shaft with modular (two parts) 3D-printed mPCL-TCP scaffolds. Following comprehensive treatment of a posttraumatic septic defect pseudarthrosis, including implantation of a hybrid fixator (Orthofix®) to support stability during infect consolidation (A), 3D reconstruction of CT imaging data showed two distinguished femoral bone defects located antero-lateral and antero-medial and also the preferred plate osteosynthesis was integrated at an early stage in the surgical planning (B). Two geometrically matched 3D-printed mPCL-TCP scaffolds loaded with ABG (C) were implanted and combined with plate osteosynthesis. Proper fit of the modular two-part scaffold of the bone defect as per the pre-operative planning (see inset in D), was confirmed intra-operatively (D).

**Fig. 7:**
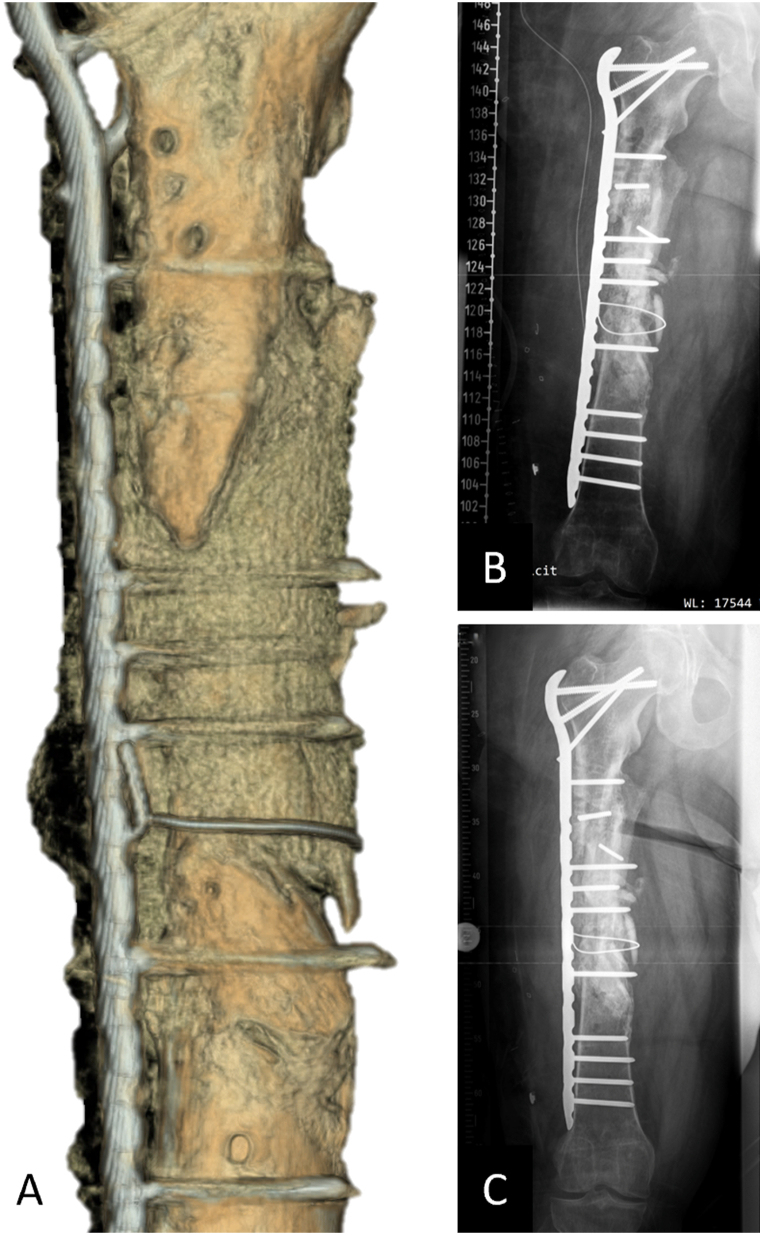
Early radiological confirmation of correct fit of patient-specific mPCL-TCP scaffolds secured with stainless steel cerclage wire. 3D reconstruction of the CT imaging seven months after scaffold implantation with early mineralisation of the fully interconnected large pore architecture (A). Further, progressing osseous consolidation five (B) and nine months (C) after implantation are shown radiographically.

This patient (42 years old) sustained a multi-fragmentary shaft fracture of the right femur due to a complex blast injury and was admitted with an infected pseudarthrosis. First, a multi-fragmentary fracture zone measuring approximately 13 ​cm and infected with multiresistant gram-negative bacteria (4 multidrug-resistant Gram-negative bacteria (MRGN)) was treated. A detailed treatment course before scaffold implantation can be found in Supplement 3. Briefly, a lateral femoral hybrid fixator (Orthofix®) was applied, and infection consolidation was performed by removing the deperiosted and infected bony fragments. Conditioning of the soft tissues was achieved with Vacuum Assisted Closure (VAC) and antibiotic therapy. At a total of 19 months after index trauma, no residual contamination with microorganisms was observed in an open biopsy. CT imaging showed signs of bone healing at the femur shaft. However, this was a complex malunion with two distinguished bone voids, requiring additional surgical treatment to achieve sufficient biomechanical stability. During preparation for scaffold implantation at previous pseudarthrosis sites, two-stage IMT and revascularization using transcortical (Pridie) drilling were performed. Two patient-specific scaffolds, each fitting a complex stand-alone bone defect, were additively manufactured and implanted in combination with RIA bone graft (harvested from the contralateral femur) and secured with a cerclage. An LCP Proximal Femoral Plate 4.5/5.0 (locking compression plate, Synthes®) was implanted to achieve additional biomechanical stability (Fig. 6). Primary wound healing was observed, and six months after implantation of the scaffold, due to radiographically diagnosed significant bony consolidation, the patient was able to bear full weight with the aid of forearm crutches (Fig. 7).

### 3.4 Case 4

**Fig. 8:**
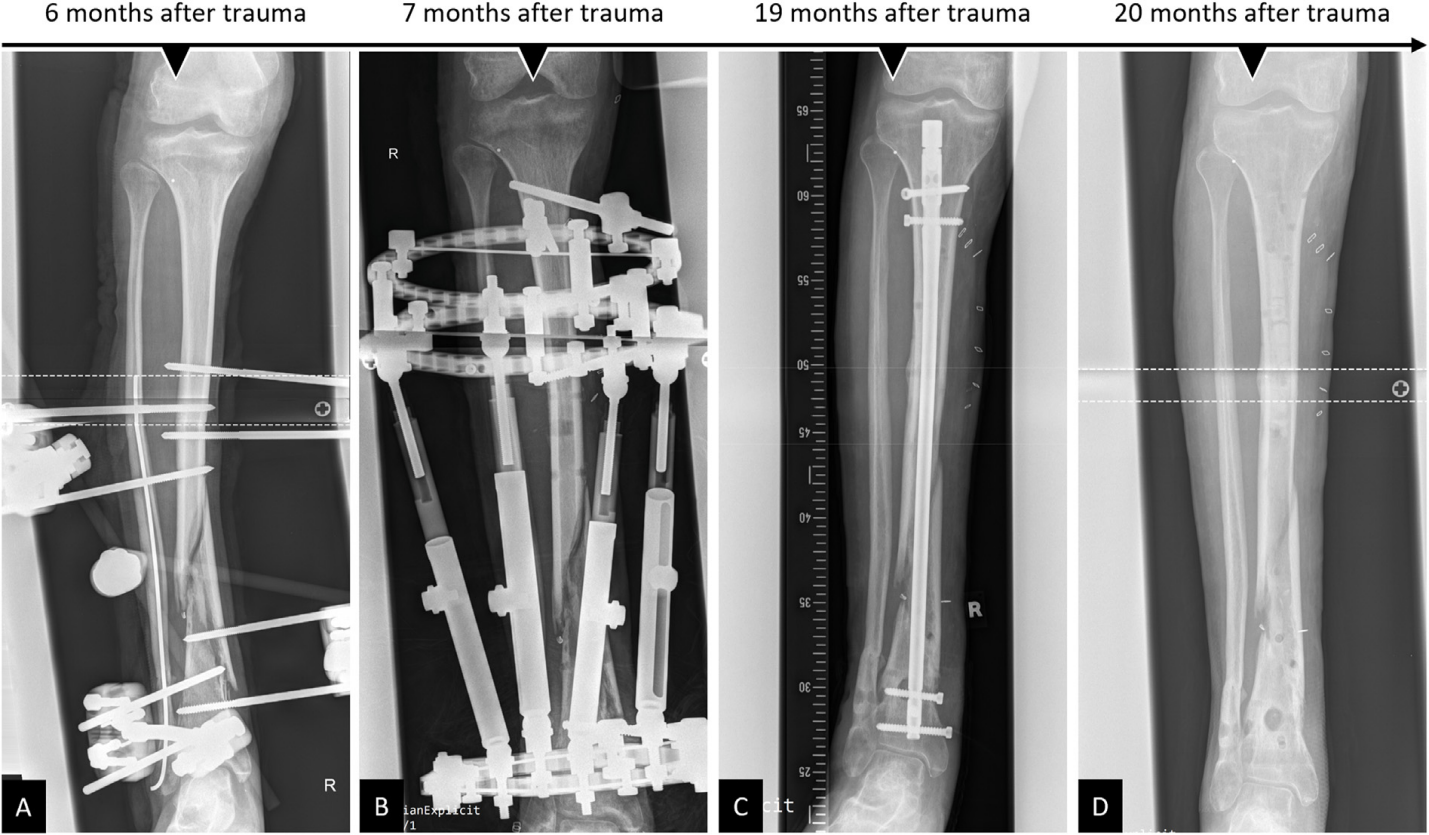
Complex course of treatment after polytrauma with distal lower leg fracture and recurrent MRSA-induced osteomyelitis. Initial treatment included open biopsy (test result: MRSA), removal of the implants (A), and implantation of an Orthofix® ring fixator (TrueLok™ Ring Fixation System) along with Cerament V® insertion in the medullary cavity of the right tibia (B). After treatment of the MRSA osteomyelitis, the procedure was changed 12 months after application of the ring fixator to a Stryker® T2 tibia nail (C). During short-term follow-up, removal of the intramedullary nail and multiple debridement due to a recurrent MRSA infection were indicated (D).

**Fig. 9:**
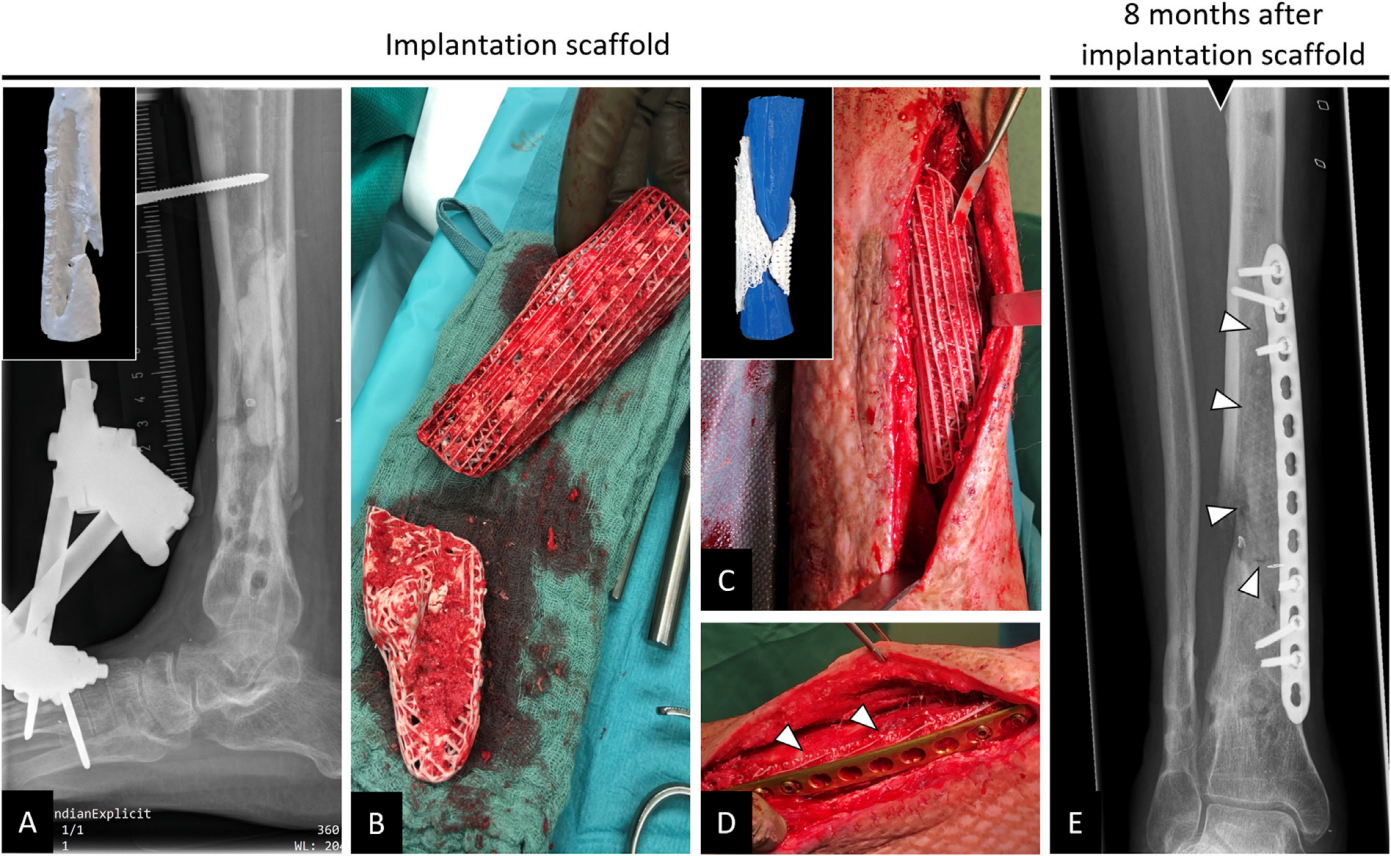
Implantation of 3D-printed patient-specific bioresorbable two-part composite scaffold in complex bone defect right distal tibia dorso-medially 22 months after index trauma. In a comprehensive course of treatment, a recurrent infected posttraumatic non-union was eventually stabilized with an external fixator with PMMA spacer (induced-membrane technique, IMT) application (A) in the defect (see inset in A for defect visualization). After persistent MRSA-induced osteomyelitis had been ruled out in an open biopsy following antibiotic therapy, a two-part mPCL-TCP scaffold fitting the irregularly shaped defect was loaded with ABG during the second stage of the IMT (B). Proper fit of the modular scaffold of the defect, as observed during pre-operative planning (see inset in C), was confirmed intra-operatively (C). The scaffold (white triangles) was additionally secured with plate osteosynthesis (D). Eight months after implantation, there was bone formation inside and outside the fully interconnected scaffold architecture (E, triangles indicate the outer border of the scaffold).

This patient (30 years old) was admitted five months after a polytrauma with multi-fragment distal lower leg fracture treated with an external fixator (tibia) and a small diameter intramedullary wire (fibula). The initial open biopsy revealed bacterial colonisation by a methicillin-resistant staphylococcus aureus (MRSA), and systematic antimicrobial treatment was initiated accordingly. A complex treatment course was followed with evidence of MRSA-induced osteomyelitis, and the external fixator was changed to an Orthofix® ring fixator (TrueLok™ Ring Fixation System) and Cerament V® (BONESUPPORT AB, Lund, Sweden) inserted into the medullary cavity. An attempt at intramedullary nail fixation 19 months after trauma resulted in early nail removal due to recurrent osteomyelitis (Fig. 8). One month after replacement with an external fixator and antibiotic-loaded Vancomycin-enriched PMMA spacer insertion to initialize an IMT, a distinct bone defect was revealed in the area of the distal dorso-medial tibia. Subsequently, after a persistent infection had been ruled out (open biopsy), a 3D-printed scaffold was used to augment the area of the former multi-fragmentary fracture zone in order to allow the patient to bear full weight in the further course of treatment. Reaming of the medullary canal and Pridie drilling at the defect sites were performed. Then, the two-part mPCL-TCP scaffold fitting into the irregularly shaped defect was loaded with iliac crest and RIA bone graft (harvested from the left proximal tibia) and carefully inserted into the Masquelet-membrane. Furthermore, Cerament V® and a 12-hole LCP 3.5 (Synthes®) were placed at the defect site. An uneventful postoperative course with primary wound healing followed, and seven months later, the patient was fully weight bearing without pain using forearm crutches for additional support (Fig. 9).

## 4 Discussion

Bone loss due to cancer or trauma is a major disease burden for millions of patients worldwide [9,46]. A review of the literature on large bone defects with or without concomitant soft tissue defects shows that 80–90% are amenable to reconstruction by conventional methods. These include limb shortening, non-vascularised autograft alone, delayed non-vascularised autograft placed into a Masquelet-membrane, distraction osteogenesis and vascularised bone with or without allograft. However, in a substantial subset of patients, these conventional reconstructive techniques are not sufficient and result in challenging to treat persistent bone defects. The field of scaffold-guided bone tissue engineering was initiated nearly three decades ago to develop alternative treatment options; options that will ideally eliminate the previously described issues of current clinically used treatment concepts. The development and study of scaffolds have seen tremendous growth over the years, with an exponentially increasing number of studies and reviews published in the PubMed database (Supplement 4). Yet, it is seldom acknowledged that in 1976 Yannas and Burke (https://www.invent.org/inductees/ioannis-v-yannas) might have been the first to define the structural features of what we define today as a “scaffold”. In a series of studies and publications, Yannas and Burke described a highly porous analogue of the extracellular matrix based on type I collagen with specific structural features [47,48]. These required features included a specific range of the pore size, defined degradation half-life and specified surface chemistry. They did not use the term “scaffold” in their work yet refer to it as the “dermis regeneration template” (DRT), as the research showed full-thickness skin wounds in animals and humans led to regeneration of a nearly physiological dermis. Yet, in the 21st century, some argue [49] that a traditional “scaffold” represents the wrong approach, and that tissue-engineering templates that are designed to exactly replicate the niche, or microenvironment, of these target cells are much likelier to succeed.

Clearly, the path of exactly replicating the niche/microenvironment has inhibited rather than enhanced SGBR largescale bench-to-bedside translation. Exact replication of the niche is not a well-thought-through idea, as the microenvironment is constantly changing in the regeneration process which takes one to three years for complex large volume bone defects as shown in the cases presented here. Furthermore, the research community frequently neglects the fact that there is often a great level of disengagement between the clinical demands of a tissue engineering concept and the scientific realisation of the concept; disconnect that hampers the clinical translation of the concept [50]. Although there is a clear and necessary role for discovery-based science in SGBR, there should be a balance between basic science and translational research and ultimately routine clinical application [51], which is not achieved when well more than 95% of SGBR studies are allocated towards (often) mediocre research just for the sake of producing publications and supporting academic careers [52]. Our research group has defined as a *conditio sine qua non* that the first step in a translation can be summarized as ‘development of an SGBR concept for a specific clinical indication and finalization of not ‘one’, but at least four to five preclinical studies in a large *in vivo* model which simulates as closely as possible the targeted human clinical indication’. In several preclinical studies, we presented evidence for our SGBR concept allowing the regeneration of large and extra-large volume segmental sheep tibia defects [[38], [39], [40], [41], [42]]. This pioneering translational research gave us the confidence to start the case series study presented in this paper.

The workflow described in this publication can be straightforwardly followed and is adaptable to a range of bone defect geometries, resulting in the formation of reproducible, dimensionally accurate, patient-specific implants. In line with a previous case report [44] the presented concept of the convergence of 3D-printed slow degrading scaffolds with ABG & rhBMPs shows the versality of SGBR to treat large to extra-large bone volumes, as in cases 1 and 2, to highly complex, irregularly shaped large defects, as in cases 3 and 4.

Moreover, very good clinical results were achieved when applied in combination with RIA bone graft in a patient with an extra-large tibial defect (case 2). Enhanced neovascularization and shorter distances for diffusion of oxygen supply and nutrients facilitated by the >70% porous scaffold might account for the beneficial biological environment, which results in less bone graft resorption, essential for functional bone regeneration [32]. Nonetheless, a large distance of the autograft from the host bone and limited soft tissue envelope are characteristic of tibial (extra-large) segmental defects, which lead to impaired blood and nutrient supply to the defect area and are ultimately associated with increased autograft resorption eventually resulting in impaired bone regeneration [53]. Therefore, evidence of successful use in both preclinical and clinical studies in improving neovascularization and nutrient transport as well as metabolic waste removal [42,44,54,55] has prompted our interdisciplinary team to include additional biological stimulation with rhBMP-2 for the optimal early bone regeneration in case 2 which had a long defect trajectory and limited tibial soft tissue coverage capacity.

Of note, contradictory results have been published regarding the preferability of implanting osteoinductive or osteoconductive graft materials inside the Masquelet-membrane during the second surgery of the IMT [24,56]. In vivo studies have shown that the Masquelet-membrane contains growth factors like vascular endothelial growth factor, transforming growth factor-beta 1 and the osteoinductive factor BMP-2 [[57], [58], [59]], as well as expressing collagen-rich matrix, and therefore might improve bone regeneration by supporting the differentiation and proliferation of mesenchymal stem cells (MSC) [60] towards the osteoblastic lineage [58]. However, overall, the osteogenic potential of IMT is very limited as the growth factor levels in the collagen membrane are insufficient to effect the proliferation and differentiation of millions of MSCs required for the regeneration of a large volume defect [61]. Furthermore, undesired creeping of the bone graft due to gravity is observed with IMT. This issue is overcome by integrating the IMT into patient-specific SGBR therapy, as the mPCL-TCP scaffold physically entraps the RIA bone graft and keeps the graft material throughout the entire length of the defect site. Furthermore, successful treatment of segmental bone loss largely depends on the defect size, because extra-large ABG volumes are difficult to harvest without significant donor side morbidity and are also associated with overly fast resorption as they are enclosed often by more than 95% soft tissue, which impairs the capacity to guide bone regeneration throughout the required period [62]. Well-designed scaffolds build a stable microenvironment that facilitates the oxygen and nutrient supply to the bone graft material. Thereby, they serve as an osteoconductive matrix, providing the structural basis for successful bony ingrowth [32] and less bone graft resorption, which in turn might reduce the volume of required ABG.

Bone healing involves a range of cells, including signalling molecules and signalling pathways that govern the healing process [63,64]. Therefore, the application of a composite biodegradable scaffold with cells seems logical; however, equipping scaffolds with stem cells is a highly demanding process with several challenges. For instance, harvesting and *ex vivo* cultivation of cells have been associated with reduced osteogenic capacity, as well as with affecting the phenotype and behaviour of these cells [65,66]. As the biodegradable material is directly coupled with a biologic, comprehensive FDA class 3 regulatory approval applies [50] and, therefore, is subject to strict regulatory control by the relevant authorities [67]. Furthermore, although progenitor cells benefit from the support of 3D-printed biodegradable scaffolds [68], their application without the support of an extracellular matrix (ECM) shows insufficient osteogenic capacity [41]. Therefore, to heal a large-sized defect, an osteoinductive and osteoconductive environment paired with biomechanical stability is required (diamond concept) [69]. In line with this, in a pre-clinical segmental defect study, mineralized cell sheets with delayed injection of allogenic stem cells providing a suitable ECM showed improved bone regeneration [39]. In particular, the ECM of ABG contains a reservoir of complete and specific structural and signalling proteins at a physiological dose and in a “non-recombinant” state [70]. Therefore, with intraoperative packing of biodegradable scaffolds with bone grafts, a highly osteogenic environment is achieved with an unquestionable capacity to regenerate large bone defects [43,44].

For diaphyseal bone defects, tubular scaffold designs are particularly suitable and have proven successful in the treatment strategy with external fixator and plate osteosynthesis (Fig. 5), as well as in the concept of nail stabilization [44]. Furthermore, irregularly shaped bone defects, such as those described in cases 3 and 4, are challenging to treat with conventional surgical methods [71]. For example, a tricortical fibula or iliac crest bone transplant is often ill fitting in an irregular osseous void. To prepare the recipient site for the tricortical bone transplant, it may be necessary to perform an osteotomy and create a segmental defect, which in turn is associated with donor site morbidities and postoperative complications. Patient-specific scaffolds for these complex, irregularly shaped large bone defects offer an alternative treatment option, and in these cases, plate osteosynthesis especially was successfully integrated into the treatment concept (Fig. 6, Fig. 9). It is important to note that especially in cases with bone defects and concomitant malunion, it is crucial to exclude bony misalignment in preoperative planning or to address its treatment in the scaffold design process, choice of internal and/or external fixation device and overall surgical planning.

To the best of our knowledge, we present the first case series of patients with posttraumatic long bone defects receiving individually tailored SGBR strategies. We reviewed the patient cases according to the proposed clinical reporting methods for medical devices for reconstruction of long bone defects [72] and observed radiographically and noninvasively appropriate limb alignment, stability and bone healing during follow-up. Moreover, full weight bearing, functional limb recovery and high patient satisfaction were achieved in all cases. However, firstly, several tightly regulated remodelling cycles involving a balance between resorption and formation are required before complete bone regeneration is achieved [73], and secondly, full resorption of mPCL-TCP scaffolds takes up to 48–60 months [74]. Therefore, in the presented patient cases, ongoing clinical follow-up is required before ultimately complete bone remodelling and thus successful completion of treatment can be confirmed. Long-term follow-up of several years with frequent check-ups is a major challenge for clinical studies in the field of these highly complex large long bone defects. Therefore, based on the promising short-to medium-term results presented in the four cases, the planning of multi-centre long-term clinical trials has been initiated.

## 5 Conclusion

This case study indicates that patient-specific SGBR represents a transformative treatment concept for large bone defects, including complex and irregularly shaped malunions. In SGBR, the scaffold design objective is not to replicate/copy the physical structure of the organ bone, but rather to guide tissue development throughout the entire regeneration process in order to facilitate physiological bone remodelling. We demonstrate that a scaffold morphology design rooted in these principles allows both the ABG and the host tissue to promote new tissue formation by providing both a surface and void space, which promotes the attachment, migration, proliferation and desired differentiation of connective tissue progenitors throughout the region where new tissue is needed.

## Authorship statement

Conception and design of study: ML, DWH, FH; acquisition of data: ML, HD, HA; analysis and/or interpretation of data: SS, BH, M-LW; drafting the manuscript: ML, DWH, HD, FH; revising the manuscript critically for important intellectual content: M-LW, HA, SS, BH; approval of the version of the manuscript to be published: ML, SS, BH, M-LW, HD, HA, DWH, FH.

## Ethical approval statement

As described in the results section the patients received mPCL-TCP scaffolds for an individual healing attempt. Since individual healing attempts are not considered human experimentation under current German law, no application to the Ethics Committee is required. However, as described in the manuscript, after receiving detailed spoken and written information, all patients explicitly provided spoken and written informed consent to agree to the suggested treatment, including the implantation of mPCL-TCP scaffolds.

## Declaration of competing interest

DWH is a cofounder and shareholder of Osteopore International Pty Ltd., a company specialized in 3D-printed bioresorbable implants to assist with bone healing. The remaining authors declare that the research was conducted in the absence of any commercial or financial relationships that could be construed as a potential conflict of interest.
