# Supplementary figures and images for: Clinical translation of a patient-specific scaffold-guided bone regeneration concept in four cases with large long bone defects
Source: J Orthop Translat. 2022 Jun 16;34:73–84. doi: 10.1016/j.jot.2022.04.004 (PMC9213234; doi:10.1016/j.jot.2022.04.004)

# Supplementary Material

## Supplement 1. Scaffold prototypes


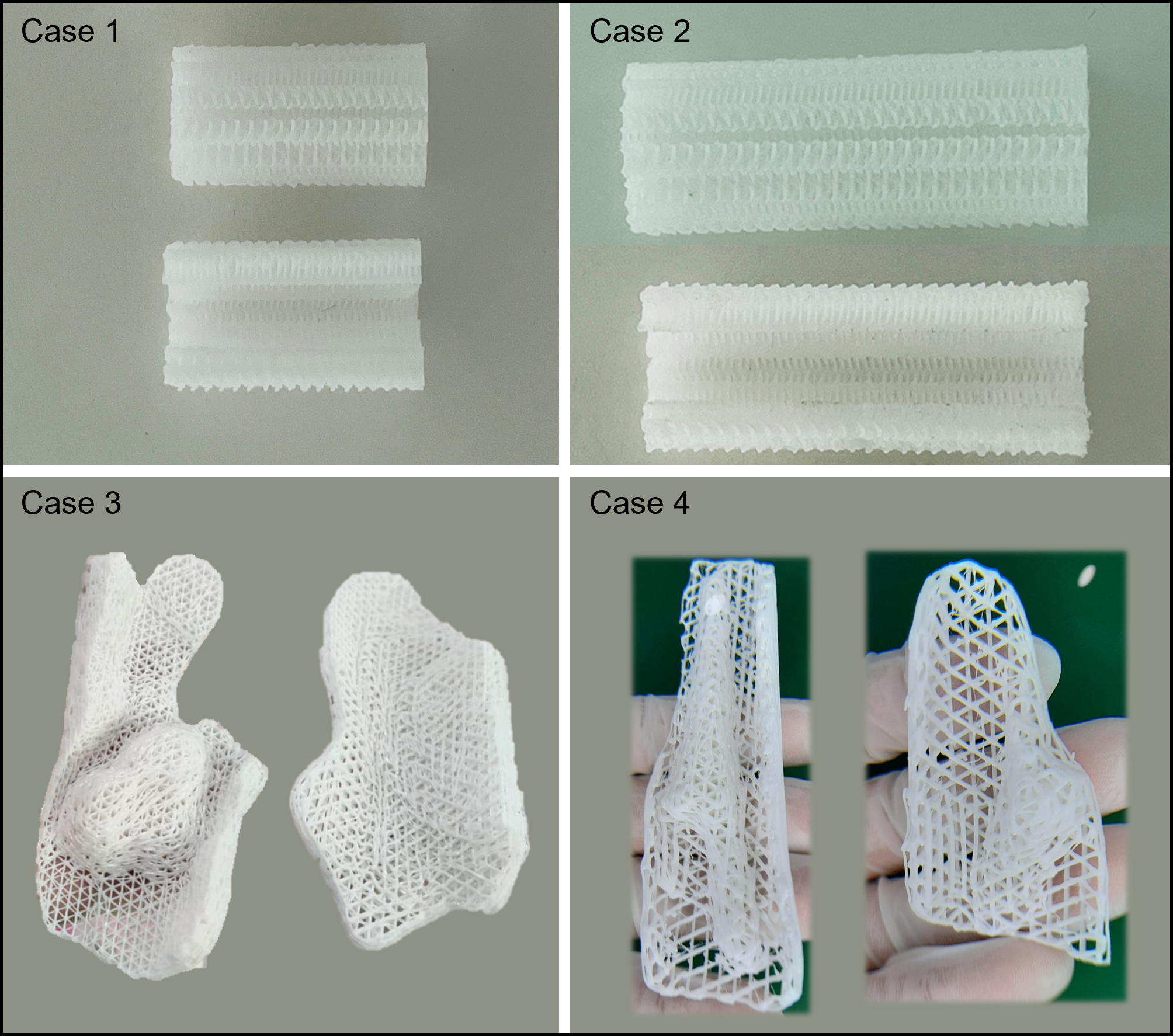

Supplement: Multimedia component 1 [file mmc1.docx]
