# Supplementary material for: Clinical translation of a patient-specific scaffold-guided bone regeneration concept in four cases with large long bone defects
Source: J Orthop Translat. 2022 Jun 16;34:73–84. doi: 10.1016/j.jot.2022.04.004 (PMC9213234; doi:10.1016/j.jot.2022.04.004)
Supplement: Multimedia component 2 [file mmc2.docx]

# Supplementary Material

## Supplement 2. Case 2 – Surgical treatment course before scaffold implantation.


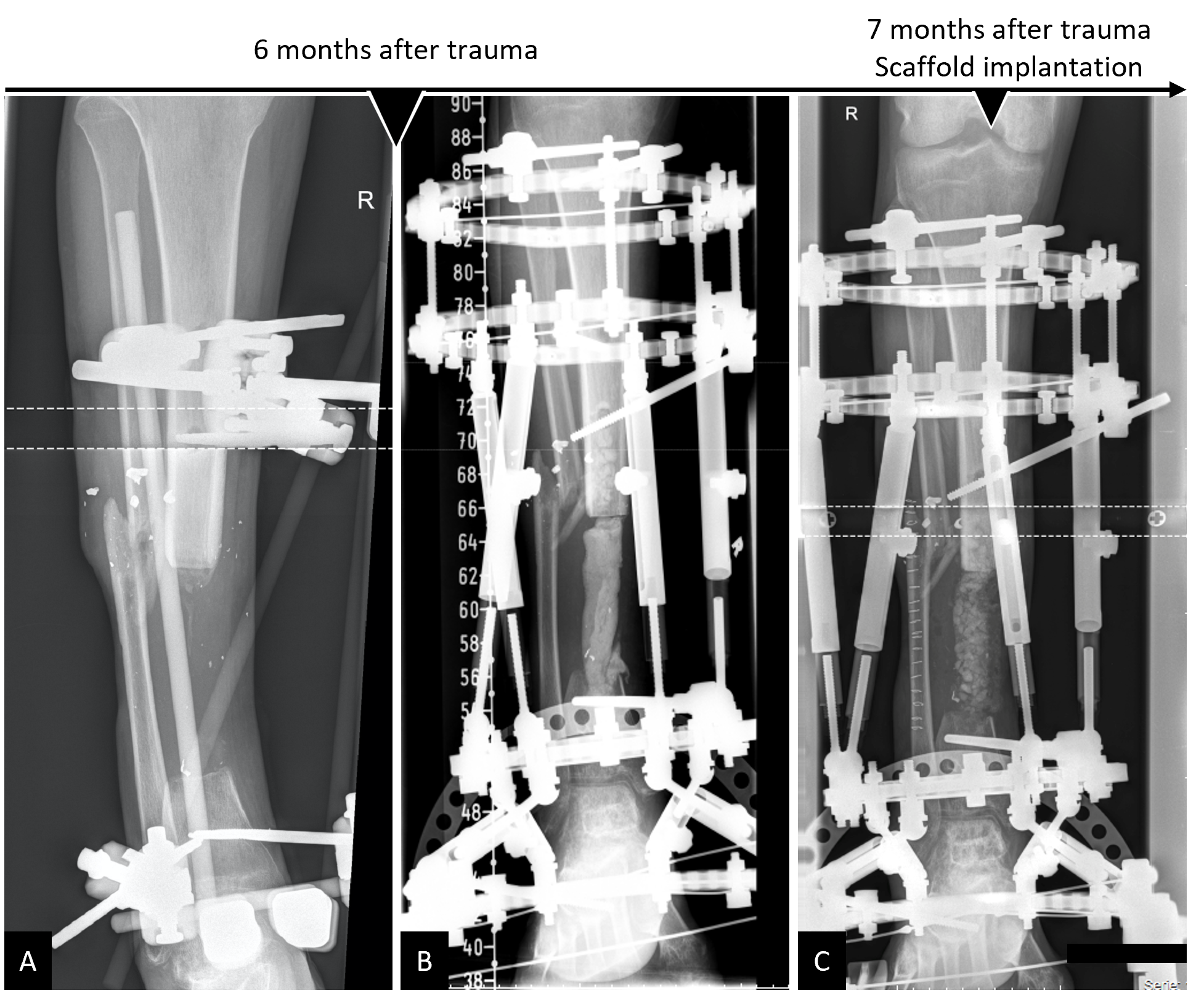


The patient was admitted six months after trauma with a 10 cm segmental defect stabilized with an external fixator (A). The induced membrane technique was initiated (B) and an external fixator was exchanged for a ring fixator Orthofix® (TrueLok™ Ring Fixation System). The mPCL-TCP scaffolds loaded with ABG and supplemented with rhBMP-2 were implanted another month later (C). ABG, autologous bone graft; mPCL-TCP, medical-grade polycaprolactone-tricalcium phosphate; rhBMP-2, recombinant human bone morphogenetic protein-2
