# Supplementary material for: Clinical translation of a patient-specific scaffold-guided bone regeneration concept in four cases with large long bone defects
Source: J Orthop Translat. 2022 Jun 16;34:73–84. doi: 10.1016/j.jot.2022.04.004 (PMC9213234; doi:10.1016/j.jot.2022.04.004)
Supplement: Multimedia component 3 [file mmc3.docx]

# Supplementary Material

## Supplement 3. Case 3 – Surgical treatment course before scaffold implantation.


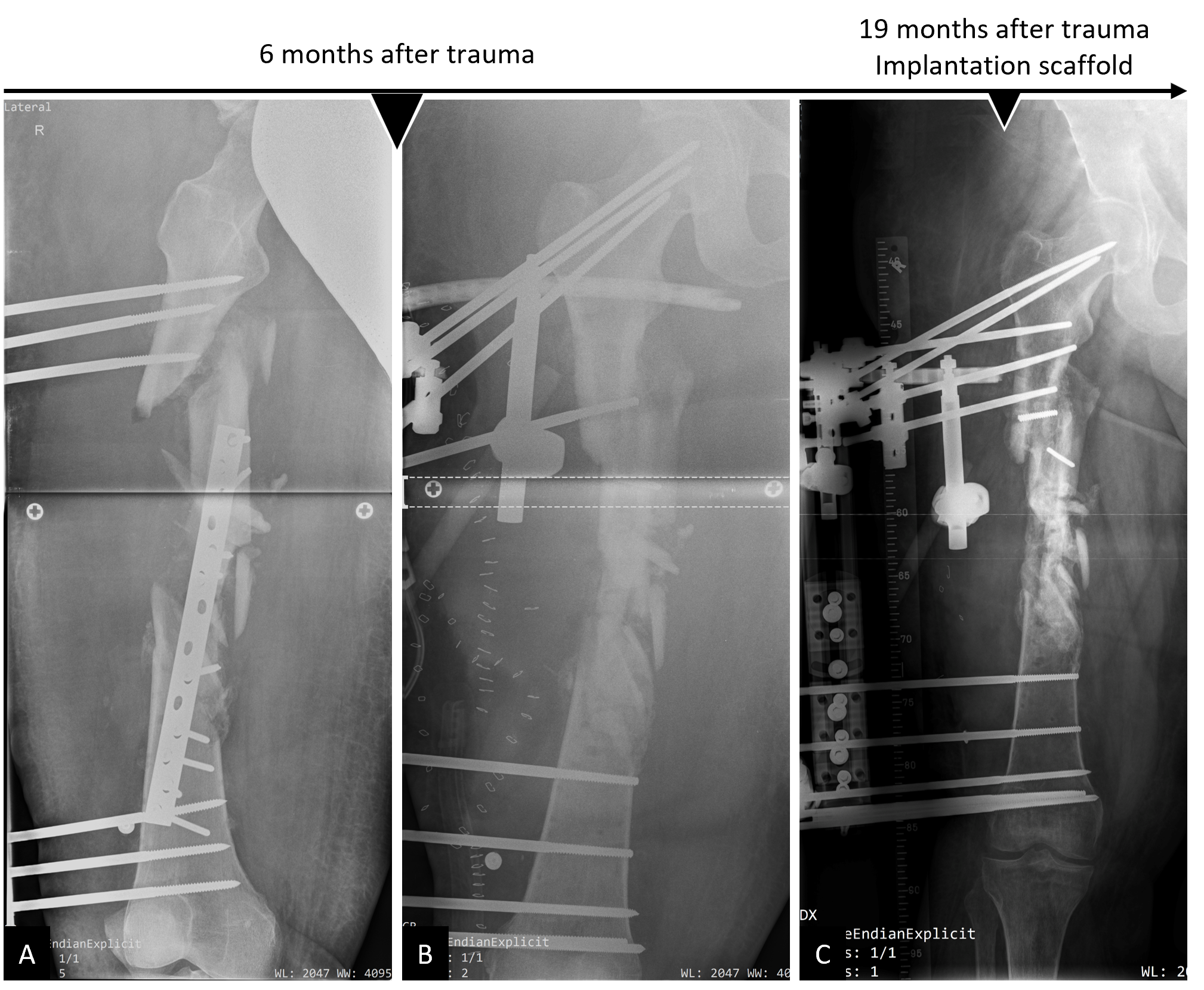


The initial revision surgery was an open biopsy with septic debridement and fistula revision (A). Microbiological analyses revealed contamination with multiple microorganisms (4 MRGN, multiresistant gram-negative bacteria), including Pseudomonas aeruginosa, Klebsiella pneumoniae, Staphylococcus epidermidis, Corynebacterium striatum and Acinetobacter baumanii. Removal of the atypically inserted large fragment plate, sequestrectomy at the femoral shaft, and exchange of the external fixator with a lateral femoral hybrid fixator (Orthofix®) as well as a Vacuum Assisted Closure (VAC) therapy with regular exchanges followed (B). At 19 months after index trauma and successful infection eradication with antibiotic and surgical treatment (C), the patient was prepared for implantation of customized composite scaffolds fitting the complex bone defect.
