# Supplementary material for: Clinical translation of a patient-specific scaffold-guided bone regeneration concept in four cases with large long bone defects
Source: J Orthop Translat. 2022 Jun 16;34:73–84. doi: 10.1016/j.jot.2022.04.004 (PMC9213234; doi:10.1016/j.jot.2022.04.004)
Supplement: Multimedia component 4 [file mmc4.docx]

# Supplementary Material

## Supplement 4. Annual growth of studies on scaffolds for bone tissue engineering published in PubMed between 1996 and 2021.


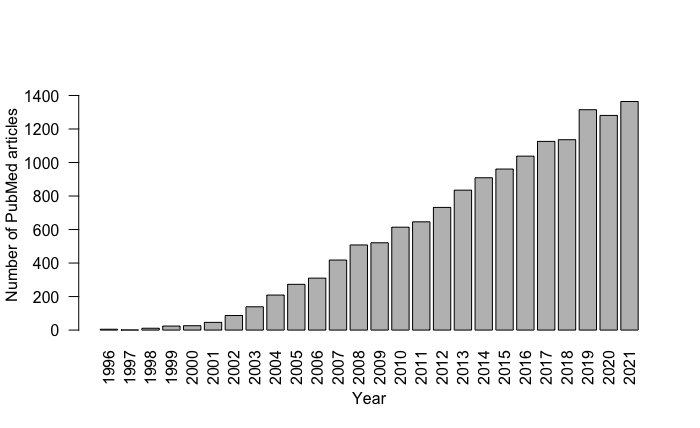


The search and bar chart illustrations were performed on 02.01.2022 using the RISmed package in R statistical software (version 4.0.2; R Foundation for Statistical Computing, Vienna, Austria) and had the following search strategy: ((engineering) AND (bone)) AND (scaffold).
